# Supplementary material for: Convergent evolution of plant pattern recognition receptors sensing cysteine-rich patterns from three microbial kingdoms
Source: Nat Commun. 2023 Jun 19;14:3621. doi: 10.1038/s41467-023-39208-8 (PMC10279758; doi:10.1038/s41467-023-39208-8)
Supplement: Supplementary file 1 — Supplementary Information [file 41467_2023_39208_MOESM1_ESM.pdf]

Supplementary Information for

Convergent evolution of plant pattern recognition receptors sensing cysteine-rich patterns from three microbial kingdoms

Yuankun Yang[1,10]*, Christina E. Steidele[1,2,10], Clemens Rössner[3], Birgit Löffelhardt[1], Dagmar Kolb[1], Thomas Leisen[4], Weiguo Zhang[1,5], Christina Ludwig[6], Georg Felix[1], Michael F. Seidl[7,8], Annette Becker[3], Thorsten Nürnberger[1], Matthias Hahn[4], Bertolt Gust[9], Harald Gross[9], Ralph Hückelhoven[2], Andrea A. Gust[1]*

This file includes:

Supplementary Figures 1 to 11

Supplementary Tables 1 to 5

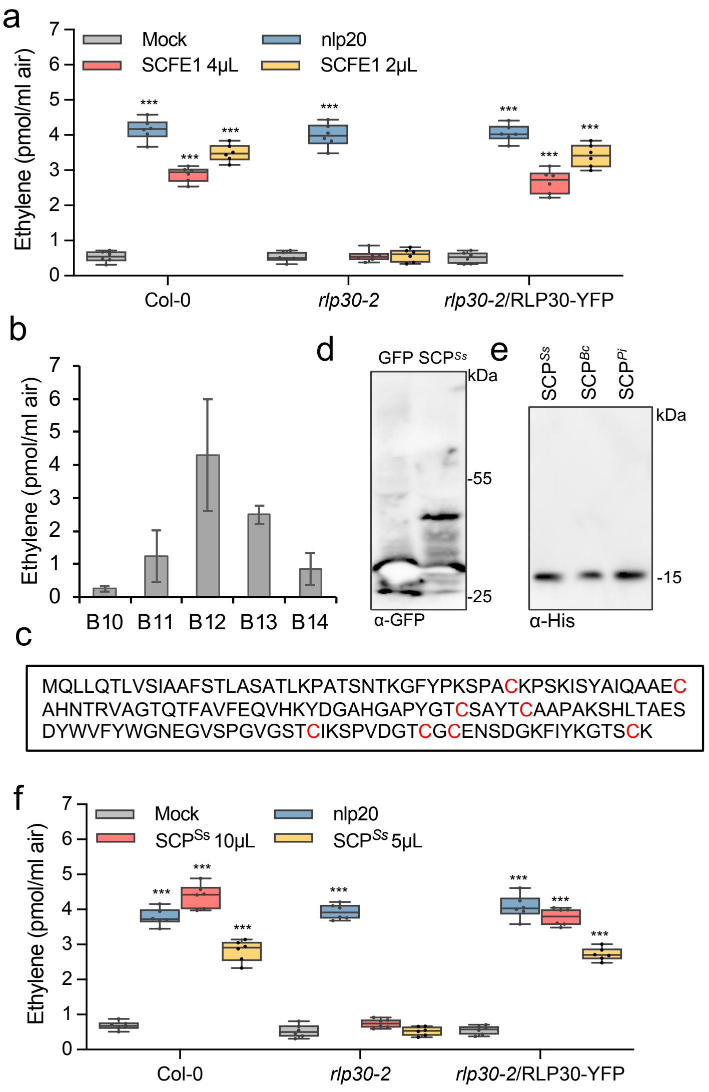

Supplementary Figure 1. **Identification of an *Arabidopsis* defense-stimulating protein in the *S. sclerotiorum* SCFE1 preparation. a** Ethylene accumulation in Col-0 wild-type plants, *rlp30-2* mutants or an *rlp30-2* line complemented with a *p35S::RL30-YFP* construct 4 h after treatment with water (Mock), 1 µM nlp20, or 2 or 4 µl per 500 µl assay volume of a SCFE1 preparation. **b** Ethylene accumulation in Col-0 wild-type plants treated with indicated SCFE1 fractions. **c** Amino acid sequence of SCP^{Ss} with the eight cysteine residues depicted in red. **d,e** Western Blot analysis of SCP from *S. sclerotiorum* (SCP^{Ss}), *B. cinerea* (SCP^{Bc}), or *P. infestans* (SCP^{Pi}) produced in the *N. benthamiana* apoplast (**d**) or purified from *P. pastoris* (**e**) using anti-GFP or anti-His antibodies, respectively. **f** Ethylene accumulation in Col-0 wild-type plants or *rlp30-2* mutants and complementation line 4 h after treatment with GFP purified from *N. benthamiana* apoplasts (Mock), 1 µM nlp20, or given volumes per 500 µl assay volume of SCP^{Ss} purified from *N. benthamiana* apoplasts. Data points are indicated as dots (*n* = 6 for a, f) and plotted as box plots (center line, median; bounds of box, the first and third quartiles; whiskers, 1.5 times the interquartile range; error bar, minima and maxima). Statistically significant differences (**a,f**) from Mock treatments in the respective plants are indicated (two-sided Student's t-test, ****P* ≤ 0.001). Source data are provided as a Source Data file. Each experiment was repeated three times with similar results.

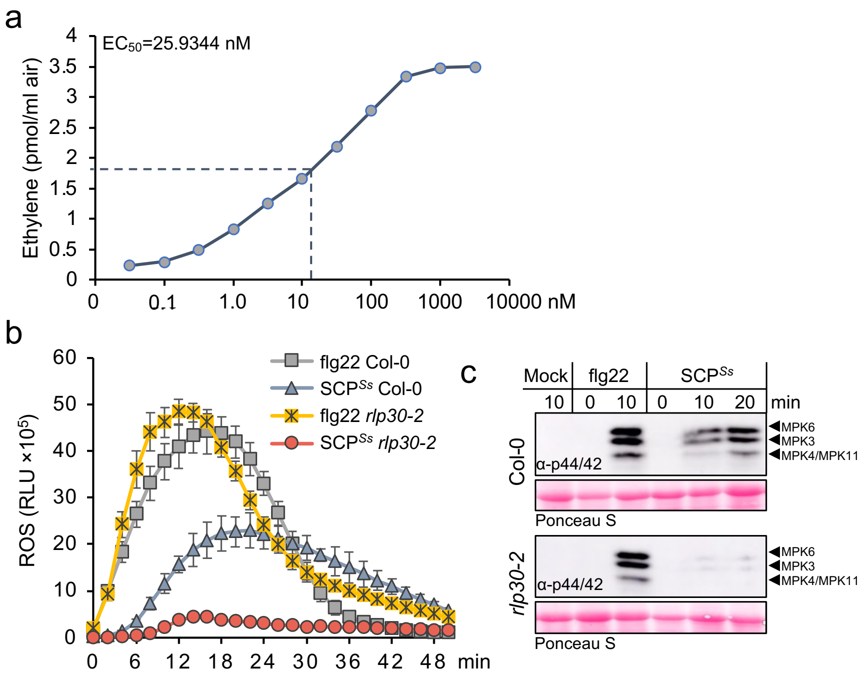

Supplementary Figure 2. **Recombinant SCP^{Ss} induces PTI responses. a** Determination of EC_{50} values using ethylene accumulation in *Arabidopsis* Col-0 wild-type plants after treatment with increasing concentrations of recombinant SCP^{Ss} (produced in *Pichia*). EC_{50} values and curve fit were calculated using EC_{50} Calculator (https://www.aatbio.com/tools/ec50-calculator). **b** ROS production in leaf pieces of *Arabidopsis* Col-0 wild-type plants or *rlp30-2* mutants treated with 0.1 µM flg22, or 2 µM SCP^{Ss}. Given are relative light units (RLU) ± SD (n = 6). **c** MAPK activation in *Arabidopsis* Col-0 wild-type plants or *rlp30-2* mutants treated for the times indicated with water (Mock), 0.1 µM flg22, or 1 µM SCP^{Ss}. MAPK activation was detected by immunoblot using phospho-p44/p42 antibodies, equal loading was verified by staining of the membrane with Ponceau S Red. Source data are provided as a Source Data file. Experiments were repeated three times with similar results.

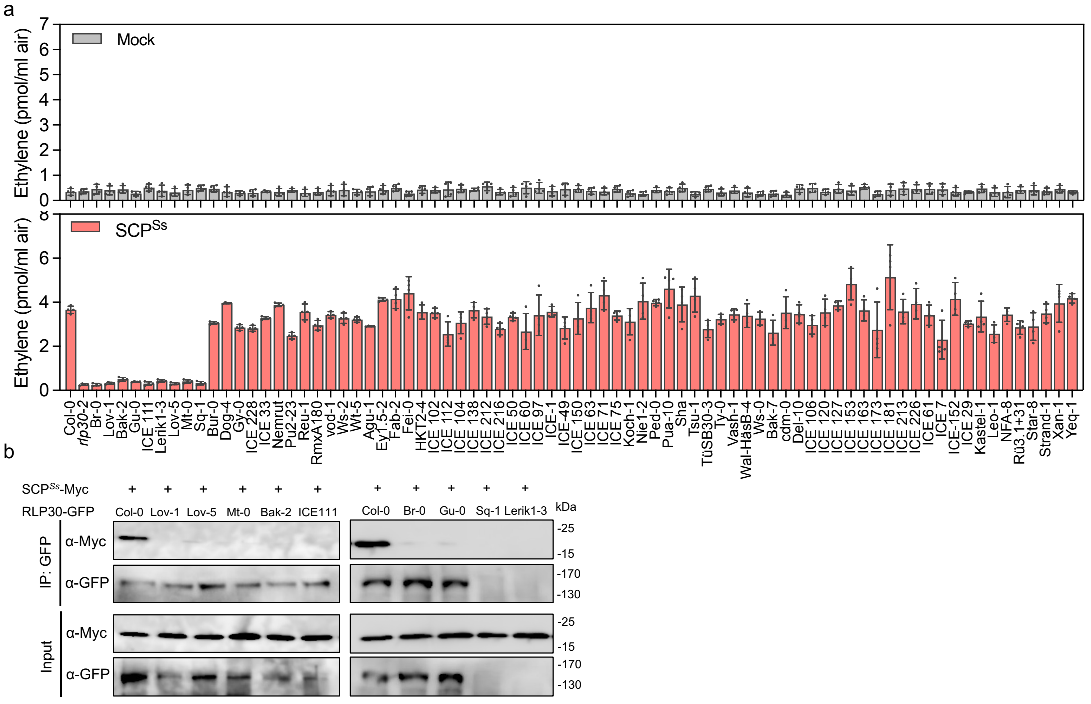

Supplementary Figure 3. **SCP^{Ss} associates with functional RLP30. a** Natural variation in SCP^{Ss} sensitivity among *Arabidopsis* accessions. 77 *Arabidopsis* accessions were tested for ethylene accumulation upon water (Mock) or SCP^{Ss} (1 μM) treatment. Data points are indicated as dots (n = 4). The experiment was repeated twice with similar results. **b** Co-immunoprecipitation assay in *N. benthamiana* transiently expressing SCP^{Ss}-myc with RLP30-GFP from different *Arabidopsis* accessions. Proteins extracted from *N. benthamiana* leaves expressing indicated protein combinations (Input) were used for co-immunoprecipitation with GFP-trap beads (IP:GFP) and immunoblotting with tag-specific antibodies. Source data are provided as a Source Data file. The experiment was repeated three times with similar results.

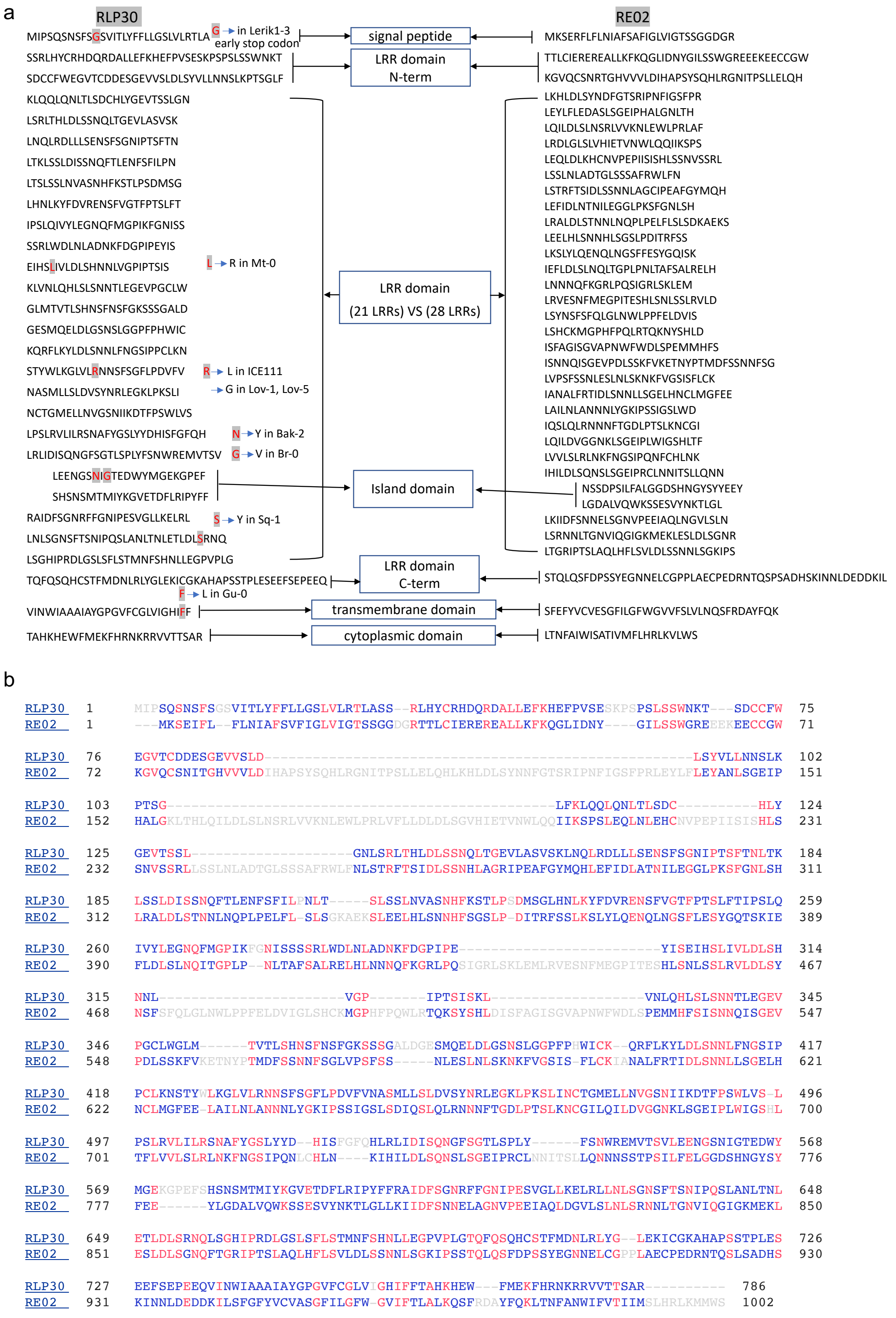

Supplementary Figure 4. **Comparison of RLP30 and RE02 protein sequences. a** Protein sequence display of RLP30 and RE02. Amino acids marked in red in the RLP30 sequence indicate amino acid changes in accessions that are insensitive to SCP^{Ss}. **b** Protein sequence alignment performed by ClustalW, conserved residues between RLP30 and RE02 are shown in red. Source Data are provided as a Source Data file.

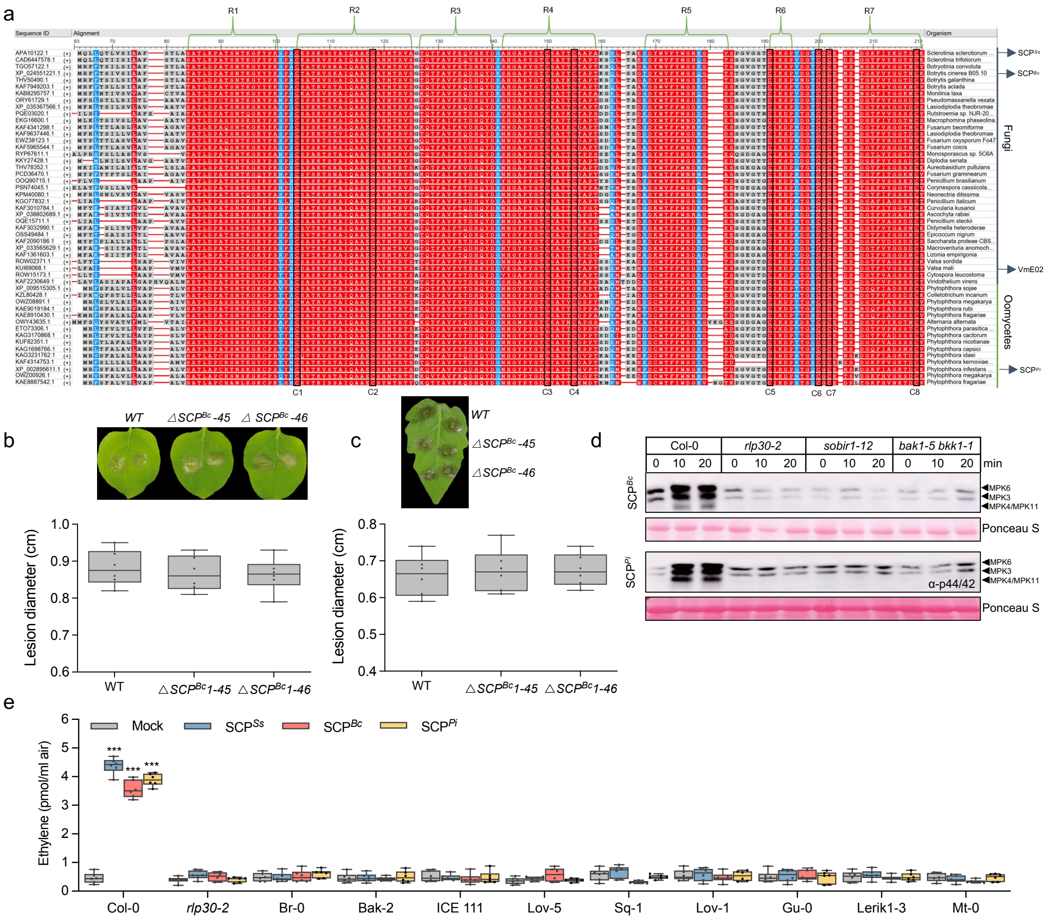

Supplementary Figure 5. **SCP^{Ss} is conserved in fungi and oomycetes. a** Amino acid alignment of SCP^{Ss} (top sequence) and its homologous sequences (Protein IDs given on the left) from the species indicated on the right. Conserved regions (in red) and conserved cysteine residues (boxed) are numbered at the top (R1 to R7) and bottom (C1 to C7), respectively. **b,c** Lesion formation of two independent *B. cinerea* depletion mutants of *SCP^{Bc}* (also named *Bcplp1*[1]) on *N. benthamiana* (**b**) or tomato (**c**) leaves 48 hours post inoculation and determination of lesion size. **d,** MAPK activation in *Arabidopsis* Col-0 wild-type plants or *rlp30-2, sobir1-12 and bak1-5 bkk1-1* mutants treated with 1 µM SCP^{Bc} or SCP^{Pi} for the times indicated. MAPK activation was detected by immunoblot using phospho-p44/p42 antibodies, equal loading of Ribulose-1,5-bisphosphate-carboxylase/oxygenase (Rubisco) was verified by staining of the membrane with Ponceau S Red. **e,** Ethylene accumulation in *Arabidopsis* wild-type plants (Col-0), or various SCP^{Ss}-insensitive accessions 4 h after treatment with water (Mock), 1 µM SCP^{Ss}, SCP^{Bc}, or SCP^{Pi}. Data points are indicated as dots (*n* = 6) and plotted as box plots (center line, median; bounds of box, the first and third quartiles; whiskers, 1.5 times the interquartile range; error bar, minima and maxima). Statistically significant differences (**e**) from Mock treatments in the respective plants are indicated (two-sided Student's t-test, ***$P \leq 0.001$). Source data are provided as a Source Data file. The experiments were repeated three times with similar results.

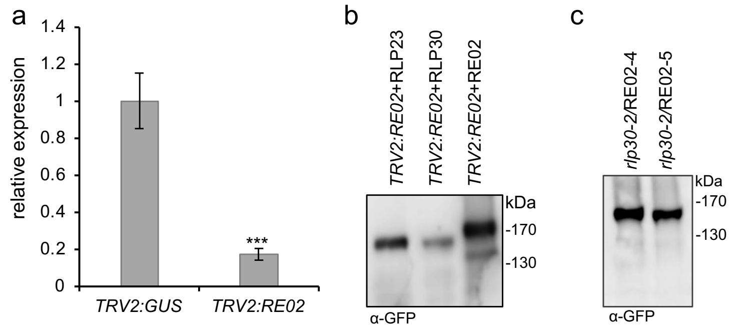

Supplementary Figure 6. **Successful silencing of RE02 and heterologous complementation. a** RT-qPCR analysis of relative expression of *RE02* in corresponding VIGS-silenced *N. benthamiana* leaves using gene-specific primers. Expression of *RE02* was normalized to the levels of *NbActin* transcript and is presented relative to the *TRV2:GUS* control which was set to 1. Statistically significant differences were determined using a two-sided Student's t-test (***$P \leq 0.001$). **b** Western Blot analysis on protein extracts from *N. benthamiana* plants silenced for *RE02* and transiently expressing GFP-tagged RLP23, RLP30, or RE02 using an anti-GFP antibody. **c,** Western Blot analysis on protein extracts from two independent *Arabidopsis rlp30-2* lines stably expressing *p35S::RE02-GFP* using an anti-GFP antibody. Source data are provided as a Source Data file.

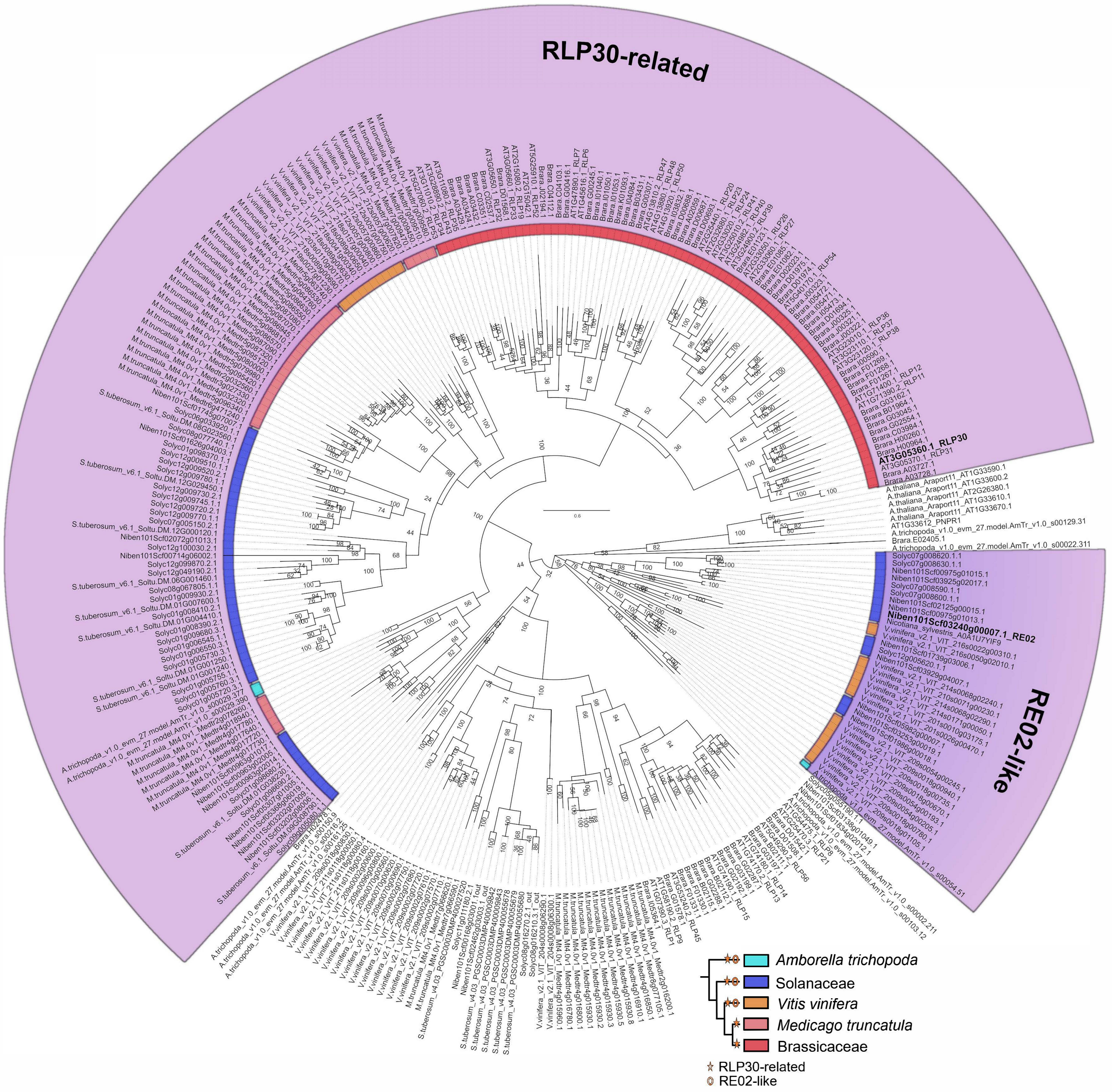

Supplementary Fig. 7. **Evolutionary relation of RLP30 and RE02.** The Midpoint rooted Maximum-likelihood phylogeny includes sequences of *A. trichopoda, S. lycopersicum, S. tuberosum, N. benthamiana, N. sylvestris, V. vinifera, M. truncatula, B. rapa* and *A. thaliana*. The least deep node that includes sequences of *A. trichopoda*, indicating independent evolution for more than 140 million years (mya), defines clades of RLP30-related and RE02-like proteins. The evolutionary history of *RLP30*-related genes is highly complex due to several lineage specific gene duplications and likely losses; since our phylogeny only covers a limited number of taxa, we named the clade that includes RLP30 as RLP30-related**.** Source data are provided as a Source Data file.

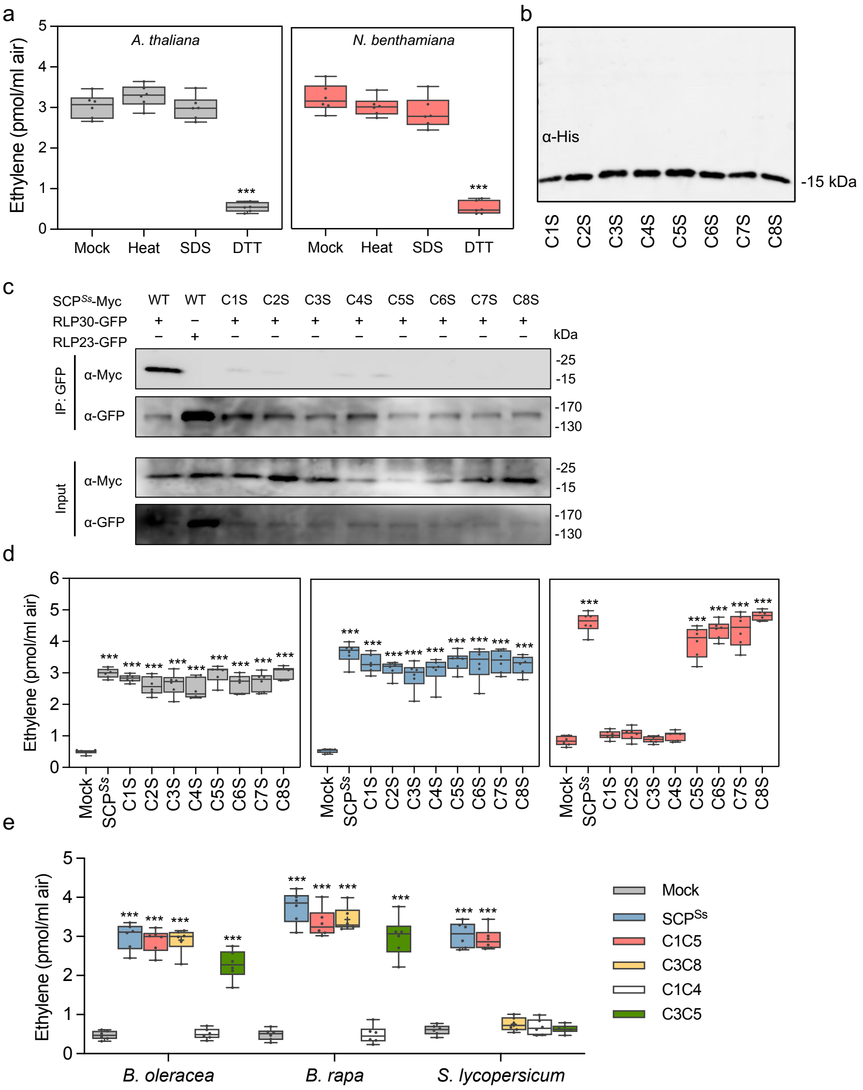

Supplementary Figure 8. **Different requirements for cysteines in SCP^{Ss} for its immunogenic activity in Brassicaceae and Solanaceae. a** Ethylene accumulation in *A. thaliana* Col-0 or *N. benthamiana* plants treated with 1 µM water-treated SCP^{Ss} (Mock), or SCP^{Ss} that was pre-treated for 1h at 95°C (heat), 1 % SDS, or 100 µM DTT. **b** Western Blot analysis of SCP^{Ss} with individual cysteine to serine mutations purified from *P. pastoris* using anti-His antiserum. **c** Co-immunoprecipitation assay in *N. benthamiana* transiently expressing SCP^{Ss}-myc and SCP-Cys mutations with RLP30-GFP or RLP23-GFP. Proteins extracted from *N. benthamiana* leaves expressing indicated protein combinations (Input) were used for co-immunoprecipitation with GFP-trap beads (IP:GFP) and immunoblotting with tag-specific antibodies. **d**. Ethylene accumulation in *Brassica oleracea*, *B. rapa,* or *Solanum lycopersicum* plants after 4 h treatment with water (Mock), SCP^{Ss}, and SCP^{Ss} with individual cysteine to serine mutations. **e** Ethylene accumulation in *B. oleracea*, *B. rapa,* or *S. lycopersicum* plants after 4 h treatment with water (Mock), SCP^{Ss}, or SCP^{Ss} truncations depicted in Fig. 3a. Data points (**a,d,e**) are indicated as dots (n = 6) and plotted as box plots (center line, median; bounds of box, the first and third quartiles; whiskers, 1.5 times the interquartile range; error bar, minima and maxima). Statistically significant differences from Mock treatments in the respective plants are indicated (two-sided Student's t-test, ***$P \leq 0.001$). Source data are provided as a Source Data file. Each experiment was repeated three times with similar results.

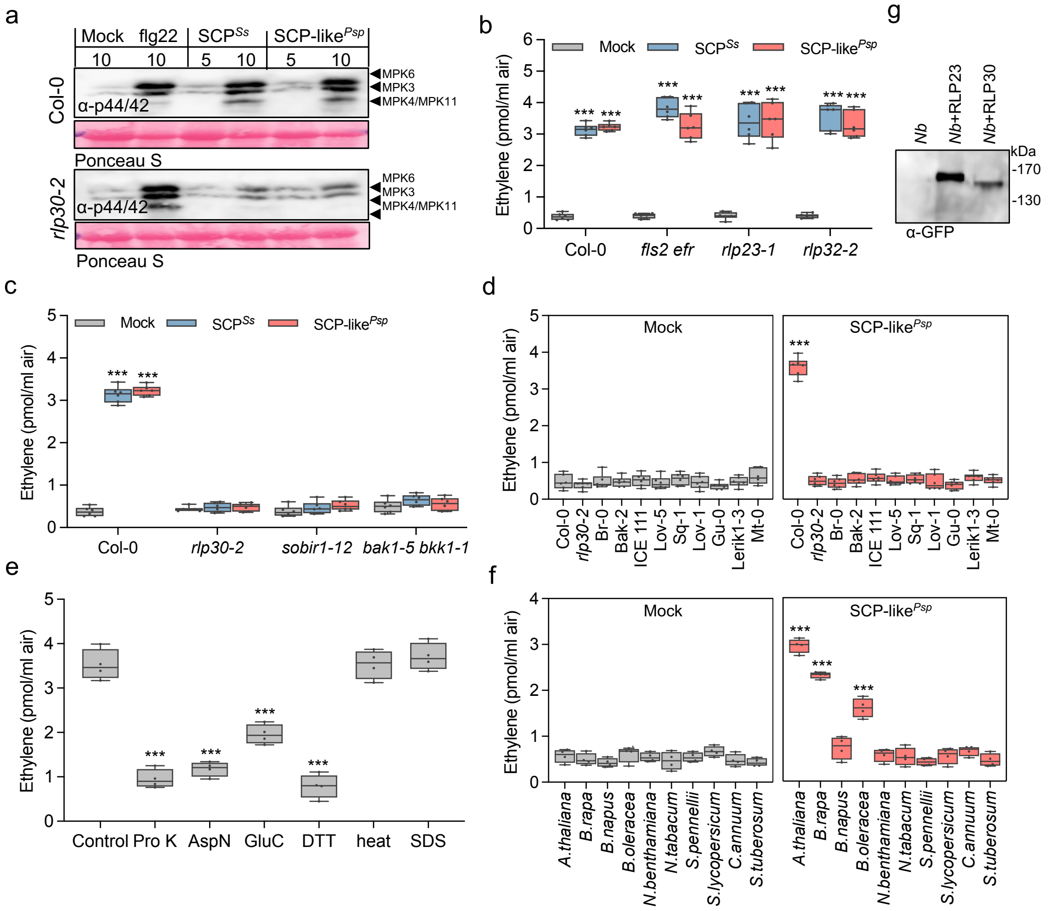

Supplementary Figure 9. ***Pseudomonas* SCP-like induces RLP30-dependent PTI responses. a** MAPK activation in *Arabidopsis* Col-0 wild-type plants or *rlp30-2* mutants infiltrated for the indicated times with 0.1 µM flg22, 1 µM SCP^{Ss}, or 1.5 µg/ml SCP-like^{Psp}. MAPK activation was detected by immunoblot using phospho-p44/p42 antibodies, equal loading was verified by staining of the membrane with Ponceau S Red. **b-d** Ethylene accumulation in Col-0 wild-type plants or indicated mutants (**b,c**) or SCP^{Ss}-insensitive accessions (**d**) 4 h after treatment with water (Mock), 1 µM SCP^{Ss}, or 1.5 µg/ml SCP-like^{Psp}. **e** Ethylene accumulation in Col-0 wild-type plants incubated for 4 h with water (Mock), or 1.5 µg/ml SCP-like^{Psp} treated for 4 h with 100 nM Proteinase K (Prot K), AspN, GluC, DTT, 1h at 95°C (heat), or 1 % SDS. **f** Ethylene accumulation in Col-0 wild-type plants or indicated plants of the Brassicaceae and Solanaceae family 4 h after treatment with water (Mock), or 1.5 µg/ml SCP-like^{Psp}. **g** Western Blot analysis on protein extracts from *N. benthamiana* leaves transiently expressing RLP30-GFP or RLP23-GFP using an anti-GFP antibody. Data points (**b-f**) are indicated as dots (*n* = 6) and plotted as box plots (center line, median; bounds of box, the first and third quartiles; whiskers, 1.5 times the interquartile range; error bar, minima and maxima). Statistically significant differences from Mock treatments in the respective plants are indicated (two-sided Student's t-test, ***$P \leq 0.001$). Source data are provided as a Source Data file. All experiments were repeated three times with similar results.

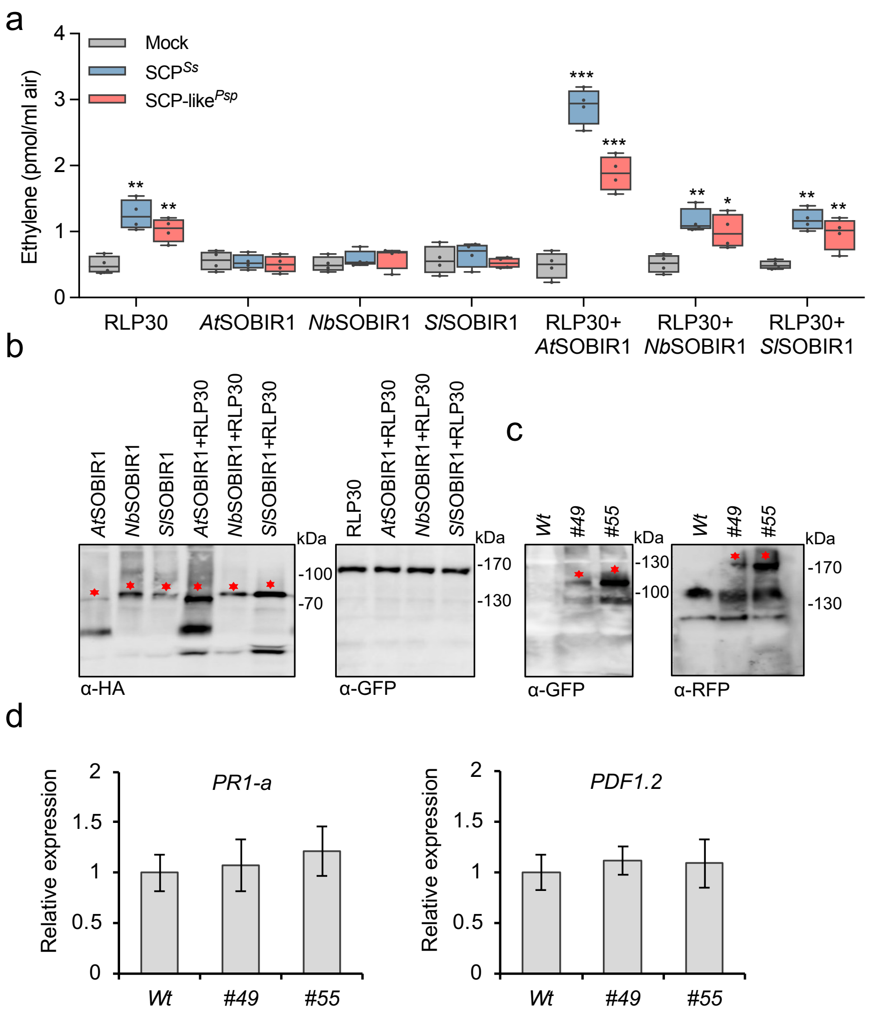

Supplementary Figure 10. **AtSOBIR1 co-expression enhances RLP30 function in *N. tabacum*. a** Ethylene accumulation in *N. tabacum* plants transiently expressing RLP30-GFP and/or SOBIR1 from *Arabidopsis* (*At*), *N. benthamiana* (*Nb*) *or S. lycopersicum* (*Sl*), either alone or in the indicated combination and treated for 4 h with water (Mock), 1 µM SCP^{Ss}, or 1.5 µg/ml SCP-like^{Psp}. Data points are indicated as dots (n = 6) and plotted as box plots (center line, median; bounds of box, the first and third quartiles; whiskers, 1.5 times the interquartile range; error bar, minima and maxima). Statistically significant differences from Mock treatments in the respective plants are indicated (two-sided Student's t-test, *$P \leq$ 0.05, **$P \leq$ 0.01, ***$P \leq$ 0.001). **b** Western Blot analysis with protein extracts from *N. tabacum* leaves transiently expressing RLP30-GFP and/or AtSOBIR1-HA, NbSOBIR1-HA or SlSOBIR1-HA as shown in (**a**) using an anti-GFP antibody for RLP30 detection and an anti-HA antibody for SOBIR1 detection. **c** Western Blot analysis with protein extracts from wild-type (Wt) plants and two independent *N. tabacum* lines (*#49* and *#55*) stably expressing RLP30-RFP and AtSOBIR1-GFP using tag-specific antisera. The asterisks indicate the position of epitope-tagged proteins. **d** RT-qPCR analysis in tobacco lines *#49* and *#55* stably expressing RLP30-RFP and AtSOBIR1-GFP using gene specific primers for indicated defense-related marker genes. Expression of marker genes was normalized to the levels of *NtACT9* transcript and are presented relative to the wild-type (Wt) control which was set to 1. Statistically significant differences were determined using a two-sided Student's t-test, differences were not significant. Source data are provided as a Source Data file. All experiments were repeated three times with similar results.

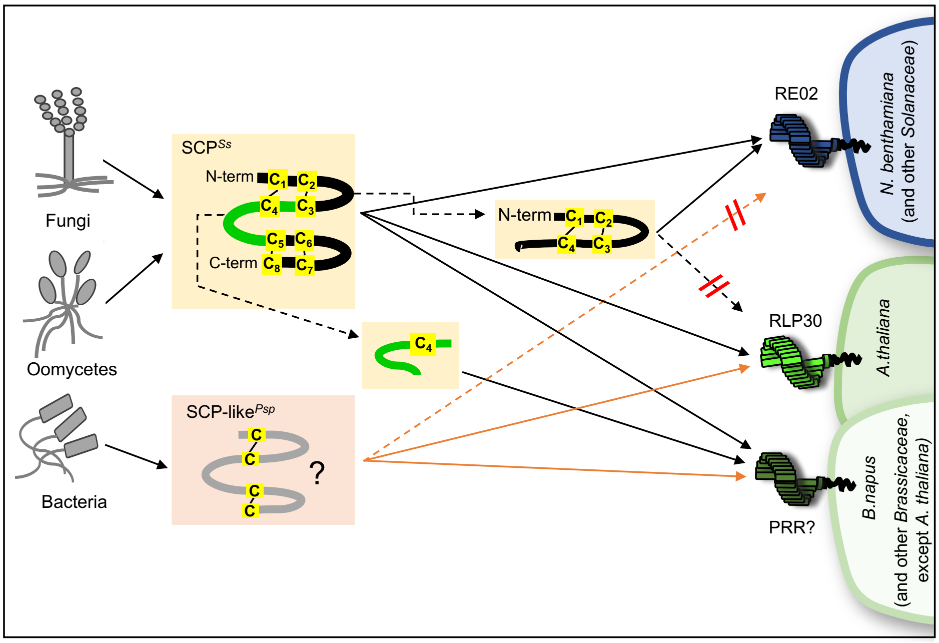

Supplementary Figure 11. **RLP30 and RE02 mediate pattern recognition.** RLP30 recognizes SCP^{Ss} and its homologs from different fungi and oomycetes, as well as an SCG1-unrelated and conserved pattern from *Pseudomonas* (SCP-like^{Psp}). RE02, an LRR-RLP non-homologous to RLP30, mediates SCP^{Ss} recognition in *N. benthamiana*. Unlike RLP30-mediated recognition of entire SCP^{Ss} in *A. thaliana*, *Brassica* species and Solanaceae perceive a small immunogenic epitope and a disulfide-bond containing peptide of SCP^{Ss}, respectively.

Supplementary Table 1. **Candidate proteins within SCFE1 obtained by MS/MS-analyses**

Protein ID	detected unique peptides per protein	Sequence coverage [%]	Mol. weight [kDa]	Sequence length [amino acid count]	signal peptide present	cysteines	Cysteine content [cysteines/seq.length in %]	MS intensity (iBAQ) in SCFE1 fraction B9	MS intensity (iBAQ) in SCFE1 fraction B11	MS intensity (iBAQ) in SCFE1 fraction B12	MS intensity (iBAQ) in SCFE1 fraction B13	MS intensity (iBAQ) in SCFE1 fraction B15
Sscle06g048920	2	17	15,481	147	yes	8	5,4	0	3349300	10808000	10764000	0
Sscle10g077860	2	18,8	21,712	191	no	1	0,5	0	1958400	626520	1260200	0
Sscle12g090490	2	6,5	26,041	245	yes	5	2	47343000	273220000	102210000	116290000	0
Sscle06g050580	2	13,4	26,089	238	yes	4	1,7	0	0	270780	1350500	0
Sscle01g009200	2	12,1	28,531	273	yes	5	1,8	769810	178500	0	0	638100
Sscle16g108160	6	19,9	34,201	327	yes	3	0,9	790000000	584600000	326300000	376090000	13573000
Sscle09g073350	2	5,3	37,229	339	yes	10	2,9	0	1079300	113530	267700	0
Sscle16g108170	3	3,7	37,672	380	yes	8	2,1	0	121680000	5940700	4476100	810830
Sscle10g080050	6	12,6	40,184	358	yes	4	1,1	525860	162740000	384270000	1745000000	730920000
Sscle14g099090	5	16,5	41,639	375	no	4	1,1	977170	1847300	1657100	1846100	3282500
Sscle03g030530	8	21,9	42,399	397	no	3	0,8	290420	303070000	151640000	695190000	24978000
Sscle04g040020	2	4,8	48,544	475	yes	11	2,3	0	3365600	2651900	4968400	0
Sscle03g028450	9	17,3	49,123	445	yes	4	0,9	0	4928200	6765900	19093000	0
Sscle02g017490	2	1,8	62,841	605	yes	8	1,3	0	747520	879350	2244900	0
Sscle11g085960	2	5,2	66,699	611	yes	6	1	0	6739000	0	10596000	0
Sscle10g080270	19	30,2	67,026	616	yes	10	1,6	12583000	245180000	423460000	570200000	16638000
Sscle08g063080	13	23,9	67,41	610	yes	8	1,3	28634000	109240000	61576000	80709000	6074600
Sscle15g106280	3	6,2	68,529	632	yes	6	0,9	583570	1179100	1547600	4099900	490000
Sscle14g100060	2	4,9	69,742	613	no	9	1,5	104900	556270	943020	2439600	1364000
Sscle06g050510	3	6,7	72,965	669	yes	9	1,3	0	242100	0	1844300	1419800
Sscle07g057170	2	5,2	99,173	907	yes	12	1,3	0	36568	0	370790	0
Sscle09g074570	4	4,2	109,64	1012	yes	2	0,2	0	1325600	1280500	1194200	0

Supplementary Table 2. ***Arabidopsis* lines used in this study**

**Line**	**Locus**	**Description**	**Reference**
bak1-5 bkk1-1	At4g33430<br>At2g13790	Double mutant of bak1-5 and SALK_044334	[2]
fls2 efr	At5g46330<br>At5g20480	Double mutant of SAIL_691_C4 and SALK_044334	[3]
rlp23-1	At2g32680	Insertion, SALK_034225	[4]
rlp30-2	At3g05360	Insertion, SALK_008911	[5]
rlp30-2/RLP30-YFP	At3g05360	Insertion, SALK_008911, complemented with YFP-tagged RLP30	This study
rlp30-2/NbRE02-GFP	At3g05360	Insertion, SALK_008911, complemented with GFP-tagged NbRE02	This study
rlp32-2	At3g05650	Insertion, SM_3_33092	[6]
sobir1-12	At2g31880	Insertion, SALK_050715, *sobir1-12*	[7]

Supplementary Table 3. **Primers used for cloning**

Template	Expression in	Primer name	Primer sequence (5' – 3')
NbRE02	*rlp30-2* *N. tabacum,* *N. benthamiana*	B_RE02-F	tatggtctcatctgaacaATGAAAAGTGAGAGATTT
		D_RE02-R	ttggtctctccttACTCCAGAGCACCTTCAATCTGTG
NbRE02	*VIGS, N. benthamiana*	TRV2:NbRE02_F	GTGAGCTCGGTACCGGATCCGAACTCCCGTCTAGTAGT
		TRV2:NbRE02_R	TGAGTAAGGTTACCGAATTCACCTTCTAGAATATTTGTATT
RLP30, all accessions	*N. tabacum,* *N. benthamiana*	RLP30-F	ATGATTCCAAGCCAATCTAATTCC
		RLP30-R(noStop)	ACGAGCACTTGTGGTGACTAC
SCP^{Ss}	*N. benthamiana* apoplast	SCP-N_F	CATTTACGAACGATAGGGTACCCCCATGCAACTCCTCCAAACCC
		SCP-N_R	TGCTCACCATGGATCCGTCGACCCCTTTACAAGAAGTCCCCTTG TAGATAAAC
SCP^{Ss} SCP^{Ss}(C1-7S)	*Pichia pastoris*	SCP-P_F	CGGAATTCATGACCCTCAAACCCGCTACCTC
		SCP-P_R	GCTCTAGACCTTTACAAGAAGTCCCCTTGTAGATAAACTTACC
SCP^{Ss}(C8S)	*Pichia pastoris*	SCP^{C8-S}-P_F	GCGGAATTCATGACCCTCAAACCCGCTACCTC
		SCP^{C8-S}-P_R	GCTCTAGACCTTTAGAAGAAGTCCCCTTGTAGATAAACTTACC
SCP^{Ss} SCP^{Ss}(C1-7-S)	*N. benthamiana*	SCP-N_F	CATTTACGAACGATAGGGTACCCCCATGCAACTCCTCCAAACCC
		SCP-N_R	TGCTCACCATGGATCCGTCGACCCCTTTACAAGAAGTCCCCTTG TAGATAAAC
SCP^{Ss}(C8S)	*N. benthamiana*	SCP^{C8-S}-_F	CATTTACGAACGATAGGGTACCCCCATGCAACTCCTCCAAACCC
		SCP^{C8-S}-_R	TGCTCACCATGGATCCGTCGACCCCTTTAGAAGAAGTCCCCTTG TAGATAAACTTACC
SCP^{Bc}	*Pichia pastoris*	SCP^{Bc}-P_F	CGGAATTCATGCTCGACCCCGCTACCTCAAAC
		SCP^{Bc}-P_R	GCTCTAGACCCTTGCAGTTCGTTCCTCCATAAACAAAC
SCP^{Pi}	*Pichia pastoris*	SCP^{Pi}-P_F	CGGAATTCATGGCTCCTTGCCGCACCAATAG
		SCP^{Pi}-P_R	GCTCTAGACCTTTGCAGTCCGTCTTGCCG
SCP^{Ss}(C1C5)	*Pichia pastoris*	SCP^{C1C5}P_F	CGGAATTCATGACCCTCAAACCCGCTACCTC
		SCP^{C1C5}P_R	GCTCTAGACCAATACAAGTCGAGCCCACACCA
SCP^{Ss}(C3C8)	*Pichia pastoris*	SCP^{C3C8}P_F	CGGAATTCATGACTTGTTCCGCTTATACCTGTGCT
		SCP^{C3C8}P_R	GCTCTAGACCTTTACAAGAAGTCCCCTTGTAGATAAACTTACC
SCP^{Ss}(C1C4)	*Pichia pastoris*	SCP^{C1C4}P_F	CGGAATTCATGGCTTGCAAACCAAGCAAAATCTCC
		SCP^{C1C4}P_R	GCTCTAGACCAGCACAGGTATAAGCGGAACAAG
SOBIR1	*N. tabacum,* *N. benthamiana*	SOBIR1-F	ATGGCTGTTCCCACGGGAAG
		SOBIR1-R(noStop)	GTGCTTGATCTGGGACAACATG

Supplementary Table 4. **Primers used for quantitative RT-PCR**

Gene	Primer name	Primer sequence (5' – 3')
NbActin	NbActin_F	TGGTCGTACCACCGGTATTGTGTT
	NbActin_R	TCACTTGCCCATCAGGAAGCTCAT
NbRE02	qRT-NbRE02_F	TGCATCCCTGAAGCCTTTGG
	qRT-NbRE02_R	TCAGGAAGTGGTTGGTTCAAGT
NtActin (ACT9)	NtACT9-F[8]	AGGGTTTGCTGGAGATGATG
	NtACT9-R[8]	CGGGTTAAGAGGTGCTTCAG
NtPDF1.2	NtPDF1.2-F[9]	GCCCTTCAATCTCTTCCAAT
	NtPDF1.2-R[9]	TATCAGGAAGACTTGCAGCG
NtPR1a/ NtPR1b/ NtPR1c	NtPR1abc-F[8]	GGATGCCCATAACACAGCTC
	NtPR1abc-R[8]	GCTAGGTTTTCGCCGTATTG

Supplementary Table 5. **SCP1 gBlocks used as PCR-templates for *Pichia* expression**

Codons for cysteines are in red, point mutations are highlighted in blue

**Name**	**Synthetic gBlock (cysteine codons in red, mutations in blue and highlighted)**
C1S	ACCCTCAAACCCGCTACCTCAAACACAAAAGGCTTCTACCCCAAATCTCCAGCTTCCAAACCAAGCAAAATC<br>TCCTACGCCATCCAAGCCGCCGAATGCGCCCACAACACCCGTGTAGCCGGCACGCAAACCTTCGCCGTCTT<br>CGAACAAGTCCACAAATATGATGGCGCACACGGTGCTCCCTATGGAACTTGTTCCGCTTATACCTGTGCTGC<br>GCCTGCGAAATCACACTTGACGGCTGAGTCTGATTATTGGGTGTTTTATTGGGGTAATGAGGGGGTTAGTCC<br>TGGTGTGGGCTCGACTTGTATTAAGAGTCCTGTGGATGGGACTTGTGGGTGTGAGAATTCGGATGGTAAGT<br>TTATCTACAAGGGGACTTCTTGTAAA
C2S	ACCCTCAAACCCGCTACCTCAAACACAAAAGGCTTCTACCCCAAATCTCCAGCTTGCAAACCAAGCAAAATC<br>TCCTACGCCATCCAAGCCGCCGAATCCGCCCACAACACCCGTGTAGCCGGCACGCAAACCTTCGCCGTCTT<br>CGAACAAGTCCACAAATATGATGGCGCACACGGTGCTCCCTATGGAACTTGTTCCGCTTATACCTGTGCTGC<br>GCCTGCGAAATCACACTTGACGGCTGAGTCTGATTATTGGGTGTTTTATTGGGGTAATGAGGGGGTTAGTCC<br>TGGTGTGGGCTCGACTTGTATTAAGAGTCCTGTGGATGGGACTTGTGGGTGTGAGAATTCGGATGGTAAGT<br>TTATCTACAAGGGGACTTCTTGTAAA
C3S	ACCCTCAAACCCGCTACCTCAAACACAAAAGGCTTCTACCCCAAATCTCCAGCTTGCAAACCAAGCAAAATC<br>TCCTACGCCATCCAAGCCGCCGAATGCGCCCACAACACCCGTGTAGCCGGCACGCAAACCTTCGCCGTCTT<br>CGAACAAGTCCACAAATATGATGGCGCACACGGTGCTCCCTATGGAACTTCTTCCGCTTATACCTGTGCTGC<br>GCCTGCGAAATCACACTTGACGGCTGAGTCTGATTATTGGGTGTTTTATTGGGGTAATGAGGGGGTTAGTCC<br>TGGTGTGGGCTCGACTTGTATTAAGAGTCCTGTGGATGGGACTTGTGGGTGTGAGAATTCGGATGGTAAGT<br>TTATCTACAAGGGGACTTCTTGTAAA
C4S	ACCCTCAAACCCGCTACCTCAAACACAAAAGGCTTCTACCCCAAATCTCCAGCTTGCAAACCAAGCAAAATC<br>TCCTACGCCATCCAAGCCGCCGAATGCGCCCACAACACCCGTGTAGCCGGCACGCAAACCTTCGCCGTCTT<br>CGAACAAGTCCACAAATATGATGGCGCACACGGTGCTCCCTATGGAACTTGTTCCGCTTATACCTCTGCTGC<br>GCCTGCGAAATCACACTTGACGGCTGAGTCTGATTATTGGGTGTTTTATTGGGGTAATGAGGGGGTTAGTCC<br>TGGTGTGGGCTCGACTTGTATTAAGAGTCCTGTGGATGGGACTTGTGGGTGTGAGAATTCGGATGGTAAGT<br>TTATCTACAAGGGGACTTCTTGTAAA
C5S	ACCCTCAAACCCGCTACCTCAAACACAAAAGGCTTCTACCCCAAATCTCCAGCTTGCAAACCAAGCAAAATC<br>TCCTACGCCATCCAAGCCGCCGAATGCGCCCACAACACCCGTGTAGCCGGCACGCAAACCTTCGCCGTCTT<br>CGAACAAGTCCACAAATATGATGGCGCACACGGTGCTCCCTATGGAACTTGTTCCGCTTATACCTGTGCTGC<br>GCCTGCGAAATCACACTTGACGGCTGAGTCTGATTATTGGGTGTTTTATTGGGGTAATGAGGGGGTTAGTCC<br>TGGTGTGGGCTCGACTTCTATTAAGAGTCCTGTGGATGGGACTTGTGGGTGTGAGAATTCGGATGGTAAGT<br>TTATCTACAAGGGGACTTCTTGTAAA
C6S	ACCCTCAAACCCGCTACCTCAAACACAAAAGGCTTCTACCCCAAATCTCCAGCTTGCAAACCAAGCAAAATC<br>TCCTACGCCATCCAAGCCGCCGAATGCGCCCACAACACCCGTGTAGCCGGCACGCAAACCTTCGCCGTCTT<br>CGAACAAGTCCACAAATATGATGGCGCACACGGTGCTCCCTATGGAACTTGTTCCGCTTATACCTGTGCTGC<br>GCCTGCGAAATCACACTTGACGGCTGAGTCTGATTATTGGGTGTTTTATTGGGGTAATGAGGGGGTTAGTCC<br>TGGTGTGGGCTCGACTTGTATTAAGAGTCCTGTGGATGGGACTTCTGGGTGTGAGAATTCGGATGGTAAGT<br>TTATCTACAAGGGGACTTCTTGTAAA
C7S	ACCCTCAAACCCGCTACCTCAAACACAAAAGGCTTCTACCCCAAATCTCCAGCTTGCAAACCAAGCAAAATC<br>TCCTACGCCATCCAAGCCGCCGAATGCGCCCACAACACCCGTGTAGCCGGCACGCAAACCTTCGCCGTCTT<br>CGAACAAGTCCACAAATATGATGGCGCACACGGTGCTCCCTATGGAACTTGTTCCGCTTATACCTGTGCTGC<br>GCCTGCGAAATCACACTTGACGGCTGAGTCTGATTATTGGGTGTTTTATTGGGGTAATGAGGGGGTTAGTCC<br>TGGTGTGGGCTCGACTTGTATTAAGAGTCCTGTGGATGGGACTTGTGGGTCTGAGAATTCGGATGGTAAGT<br>TTATCTACAAGGGGACTTCTTGTAAA
C8S	ACCCTCAAACCCGCTACCTCAAACACAAAAGGCTTCTACCCCAAATCTCCAGCTTGCAAACCAAGCAAAATC<br>TCCTACGCCATCCAAGCCGCCGAATGCGCCCACAACACCCGTGTAGCCGGCACGCAAACCTTCGCCGTCTT<br>CGAACAAGTCCACAAATATGATGGCGCACACGGTGCTCCCTATGGAACTTGTTCCGCTTATACCTGTGCTGC<br>GCCTGCGAAATCACACTTGACGGCTGAGTCTGATTATTGGGTGTTTTATTGGGGTAATGAGGGGGTTAGTCC<br>TGGTGTGGGCTCGACTTGTATTAAGAGTCCTGTGGATGGGACTTGTGGGTGTGAGAATTCGGATGGTAAGT<br>TTATCTACAAGGGGACTTCTTCTAAA

Supplementary References

1 Leisen, T. *et al.* Multiple knockout mutants reveal a high redundancy of phytotoxic compounds contributing to necrotrophic pathogenesis of Botrytis cinerea. *PLoS pathogens* **18**, e1010367, doi:10.1371/journal.ppat.1010367 (2022).

2 Schwessinger, B. *et al.* Phosphorylation-dependent differential regulation of plant growth, cell death, and innate immunity by the regulatory receptor-like kinase BAK1. *PLoS Genet* **7**, e1002046, doi:10.1371/journal.pgen.1002046 (2011).

3 Nekrasov, V. *et al.* Control of the pattern-recognition receptor EFR by an ER protein complex in plant immunity. *EMBO J* **28**, 3428-3438, doi:10.1038/emboj.2009.262 (2009).

4 Albert, I. *et al.* An RLP23-SOBIR1-BAK1 complex mediates NLP-triggered immunity. *Nature plants* **1**, 15140, doi:10.1038/nplants.2015.140 (2015).

5 Zhang, W. *et al.* Arabidopsis receptor-like protein30 and receptor-like kinase suppressor of BIR1-1/EVERSHED mediate innate immunity to necrotrophic fungi. *Plant Cell* **25**, 4227-4241, doi:10.1105/tpc.113.117010 (2013).

6 Fan, L. *et al.* Genotyping-by-sequencing-based identification of Arabidopsis pattern recognition receptor RLP32 recognizing proteobacterial translation initiation factor IF1. *Nat Commun* **13**, 1294, doi:10.1038/s41467-022-28887-4 (2022).

7 Gao, M. *et al.* Regulation of cell death and innate immunity by two receptor-like kinases in Arabidopsis. *Cell host & microbe* **6**, 34-44, doi:10.1016/j.chom.2009.05.019 (2009).

8 Riviere, M. P., Marais, A., Ponchet, M., Willats, W. & Galiana, E. Silencing of acidic pathogenesis-related PR-1 genes increases extracellular beta-(1->3)-glucanase activity at the onset of tobacco defence reactions. *J Exp Bot* **59**, 1225-1239, doi:10.1093/jxb/ern044 (2008).

9 Jin, W. *et al.* NtWRKY-R1, a Novel Transcription Factor, Integrates IAA and JA Signal Pathway under Topping Damage Stress in Nicotiana tabacum. *Front Plant Sci* **8**, 2263, doi:10.3389/fpls.2017.02263 (2017).
